# Brewers’ Spent Grain-Derived Arabinoxylan as a Sustainable Filler for Enhanced PHBV Biocomposites

**DOI:** 10.3390/polym17010114

**Published:** 2025-01-05

**Authors:** Ilary Belardi, Fabrizio Sarasini, Jacopo Tirillò, Pietro Russo, Giovanni De Francesco, Ombretta Marconi, Assunta Marrocchi

**Affiliations:** 1 Department of Agricultural, Food and Environmental Sciences, University of Perugia, 06121 Perugia, Italy; ilary.belardi@dottorandi.unipg.it (I.B.); giovanni.defrancesco@unipg.it (G.D.F.); ombretta.marconi@unipg.it (O.M.); 2 Department of Chemical Engineering Materials Environment and UdR INSTM, University of Rome La Sapienza, 00184 Rome, Italy; jacopo.tirillo@uniroma1.it; 3 Institute of Polymers, Composites and Biomaterials, National Research Council, 80078 Pozzuoli, Italy; pietro.russo@ipcb.cnr.it; 4 Italian Brewing Research Centre (CERB), University of Perugia, 06126 Perugia, Italy; 5 Department of Chemistry, Biology and Biotechnology, University of Perugia, 06123 Perugia, Italy

**Keywords:** polyhydroxybutyrate-valerate (PHBV), arabinoxylan, brewers’ spent grain (BSG), biocomposites, sustainability

## Abstract

Poly(3-hydroxybutyrate-co-3-hydroxyvalerate) (PHBV) is a highly promising biodegradable and bio-based thermoplastic recognized for its environmental benefits and potential versatility. However, its industrial adoption has been limited due to its inherent brittleness and suboptimal processability. Despite these challenges, PHBV’s performance can be tailored for a wide range of applications through strategic modifications, particularly by blending it with other biodegradable polymers or reinforcing it with natural fibers and bio-based fillers. This study explores the potential of brewers’ spent grain (BSG) as a sustainable source for the development of PHBV biocomposites. The biocomposites were synthesized by incorporating arabinoxylan-bound benzoate, which can be derived from BSG, as a sustainable filler at concentrations of 4% and 10% *w*/*w*. The resulting materials were characterized using tensile testing, scanning electron microscopy (SEM), thermogravimetric analysis (TGA), and differential scanning calorimetry (DSC). The findings demonstrate that the incorporation of functionalized arabinoxylan significantly enhances the mechanical properties of PHBV, preserves its thermal stability, and increases its crystallinity (from 59.9% to 67.6%), highlighting a positive impact on both material performance and processing characteristics.

## 1. Introduction

Plastics are ubiquitous in modern society, providing numerous benefits that make them an essential part of daily human activities. However, the extensive use of plastics has led to significant environmental and health challenges. Each year, 438 Mt of plastic waste is generated globally [1]. The accumulation and fragmentation of this waste in oceans, inland waters, and terrestrial ecosystems have become increasingly problematic, posing a growing threat to biodiversity and human well-being [2]. As the global population expands, plastic consumption continues to rise, yet efficient disposal and recycling systems remain inadequate in many regions. This trend has amplified the urgency to adopt strategies such as reducing, reusing, and recycling existing plastics while simultaneously advancing sustainable alternatives to fossil-based plastics. In response, the European Union has implemented several regulatory policies, many of them related to the European Green Deal, including the Single-Use Plastics Directive 2019/904, the Plastics strategy (2018), the new Circular Economy Action Plan (2020), and the Packaging and Packaging Waste Regulation (review 2022), which aim to reduce plastic consumption, promote recycling, and facilitate the transition to more sustainable alternatives [3,4,5,6]. Among these alternatives, biodegradable and/or bio-based plastics—commonly referred to as “bioplastics”—have emerged as a promising solution. However, bioplastics still account for only 0.7% of the more than 400 million tonnes of plastic produced globally each year [1]. Among the various types of bioplastics, polyhydroxyalkanoates (PHAs) stand out as a promising alternative to conventional fossil-based plastics [7,8]. PHAs are a family of bio-based, biodegradable, and non-toxic polymers with thermoplastic properties, naturally produced by bacteria [9,10,11]. According to the European Bioplastics update in 2024, PHAs represented 4.1% of bioplastic production, following poly(lactic acid) (PLA) and poly(butylene adipate-co-terephthalate) (PBAT) at 31.0% and 4.6%, respectively [12]. By 2029, PHAs’ production capacity is expected to grow to 17%, underscoring their increasing significance in the transition toward more sustainable materials [12].

A notable example is poly(3-hydroxybutyrate-co-3-hydroxyvalerate) (PHBV). PHBV is recyclable, industrially compostable, and suitable for a range of applications, including packaging, reinforced materials, and biomedical uses such as drug delivery and tissue engineering [13], exhibiting superior physical properties, such as enhanced toughness and processability, compared to other PHA homo-polyesters. In addition, PHBV-based materials have been approved for food contact applications under the European Plastics Regulation (EU) No 10/2011, with their inert behavior in liquid food simulants confirmed through overall migration testing [14,15]. In addition, Bonnenfant et al. evaluated the structural evolution of PHBV-based materials (both pure and loaded with 1 wt.% quercetin) over multiple cycles to assess their reusability as food packaging [14]. The PHBV material showed minimal changes in structural and physicochemical properties when exposed to 10% ethanol and 3% acetic acid, with migration levels below the EU limit of 10 mg/dm^2^ [14]. After washing and renewed contact, migration dropped to zero, and structural integrity was preserved. However, repeated cycles can alter the surface structure and increase migration. The material endured up to 20 washing cycles with water and detergent without significant changes, and even a 1 wt.% NaOH wash, which removed the surface layer, left its physicochemical properties intact [14]. However, its brittleness and process costs remain significant challenges, limiting its widespread use.

To address the property limitations, extensive research has focused on developing biocomposites, where the incorporation of fillers enhances existing properties or introduces entirely new features. Various natural fillers have shown potential for improving PHBV [16], including wheat straw [17], cellulose fiber [18], cellulose nanocrystals [19], and chitosan [20]. This study aims to explore the potential of brewers’ spent grain (BSG) in enhancing the properties of PHBV. BSG is the primary by-product generated during beer production, accounting for approximately 85% of the total by-products produced in the brewing process [21]. Its abundance, year-round availability, and rich composition of fibers (~60%) make BSG an attractive and sustainable source for various applications. For every 100 L of beer produced, nearly 20 kg of wet BSG is generated. This translates into a massive global annual production of around 40 million tons, with Europe contributing approximately 8 million tons [22,23].

While previous studies have investigated BSG as a filler material [24,25,26,27,28,29,30], the results have often been limited in promise [22]. This study aims to further explore the potential of BSG by focusing on one of its key components: arabinoxylans (AXs). AXs constitute approximately 17% of the BSG composition [31]. They are complex non-starch polysaccharides with a backbone consisting of repeating β-D-xylopyranoside (xylose) units linked through β-(1→4) bonds, and branching arabinose side chains (α-L-arabinofuranosyl residues) attached to the xylose units at the O-2 or O-3 positions via α-(1→3) and/or α-(1→4) bonds [22,32,33,34]. Additionally, hydroxycinnamic acids, such as p-coumaric and ferulic acids, can be ester-linked at the O-5 position of the α-L-arabinofuranosyl residues. The degree of substitution in AX structures can vary, influencing their functional properties, solubility, and viscosity [22,32,33,34].

Some of us have recently patented a method for the fractionation of BSG, envisaging a step of simultaneous extraction and functionalization of AXs from BSG [31,35]. Leveraging this approach, a benzoate-functionalized AX derivative was synthesized, specifically designed to serve as a bio-based filler for PHBV composites. The rationale for this choice lies in the benzoate modification, which is anticipated to increase the hydrophobicity of AXs, thereby enhancing its compatibility with the PHBV matrix and fostering stronger filler–matrix interactions. Incorporating the functionalized AXs into PHBV is expected to produce synergistic effects that mitigate the polymer’s inherent brittleness and suboptimal mechanical properties, all while maintaining its thermal stability. The development of these composite formulations aligns with recent research on new food packaging materials to substitute synthetic plastics. In particular, natural polysaccharides as food packaging materials are increasingly attracting the attention of researchers due to their abundance, sustainability, environmental friendliness, low toxicity, biodegradability, and biocompatibility. Worth mentioning are starch, gelatin, cellulose, chitosan, sodium alginate, and their derivatives [36,37,38]. Their poor water vapor barrier performance and mechanical properties can be improved by blending them with biodegradable materials and adding inorganic particles, as demonstrated by Wang et al., who prepared bio-based films incorporating carboxymethyl chitosan–ZnO nanoparticles and sodium alginate in a multilayer film with chitosan film as the outer layer by solution casting [37]. This film exhibited good mechanical properties (up to 24.4 MPa) but at the expense of a complex architecture produced through a process that is difficult to scale industrially.

This work presents an investigation into the effects of varying contents of the benzoate-functionalized AX derivative on the mechanical properties, morphology, and thermal characteristics of PHBV films manufactured using traditional plastic processing equipment.

## 2. Materials and Methods

### 2.1. Materials

Brewers’ spent grains were collected from the production of a pale ale beer (pilsner barley malt) at the brewing pilot plant of the Italian Brewing Research Centre (CERB), University of Perugia (Perugia, Italy). BSGs were stored frozen (−80 °C) in the dark to prevent their deterioration until utilization. Medium-viscosity standard arabinoxylan (~22 cSt.; P.M. 323,000) was purchased from Megazyme (Megazyme International, Wicklow, Ireland). The benzoate-functionalized arabinoxylan from the standard material (B-AX(STD)) was synthesized using a previously reported functionalization protocol [31]. The benzoate-arabinoxylan from BSG (B-AX(BSG)) was obtained using a patented BSG fractionation process, as detailed in previous work [31,35] and in the Supplementary Information. A commercial grade of the biopolymer poly(3-hydroxybutyrate-co-3-hydroxyvalerate) (PHBV), ENMAT Y1000P^®^, was purchased from the manufacturer TianAn-ENMAT (Ningbo City, China).

All additional chemicals employed in this work were purchased from Merck KGaA (Darmstadt, Germany) and used without further purification, unless otherwise noted.

### 2.2. Biocomposite Formulations

A two-step process consisting of extrusion and compression molding was used to produce the biocomposite films. Neat PHBV polymer was extruded to produce a control sample, which served as the baseline. Different formulations of PHBV with B-AX(STD) or B-AX(BSG) at two concentrations (4% and 10% *w*/*w*) were produced. Initially, a weight percentage of 10% was chosen in an attempt to minimize the amount of polymer as much as possible. However, the preliminary results showed a significant reduction in the ductility of the composite, making it excessively brittle and poorly processable for the intended application in the packaging sector. At this point, the decision was made to reduce the percentage to 4% by weight in order to limit the brittleness of the polymer without compromising its processability. The extrusion of the different formulations was performed using a Process 11 Parallel Twin-Screw Extruder (Thermo Fisher Scientific™, Waltham, MA, USA) set to 8 different temperatures in the various zones of the extruder (i.e., 150, 155, 160, 165, 170, 175, 175, and 175 °C). A screw speed of 100 rpm and a feed rate (via a single-screw volumetric feeder) of 5 were used. After extrusion, the pellets were pressed using a Platen Press P400 E (COLLIN Lab & Pilot Solutions GmbH, Maitenbeth, Germany). Pellet molding was initially carried out by drying the pellets in a vacuum oven (80 °C, 3 h). Next, molding was performed at 180 °C with a pressure cycle (2 min, 0 bar; 1 min, 10 bar; 1 min, 20 bar; 1 min, 40 bar). Finally, the biocomposite films were cooled to room temperature with a constant pressure of 40 bar. The thickness of the bioplastic films was measured with a micrometer. The films produced were named as follows:

- PHBV: the neat PHBV;
- PHBV/4 B-AX(BSG): the biocomposite films produced with PHBV and 4% (*w*/*w*) of functionalized arabinoxylan from the standard;
- PHBV/10 B-AX(BSG): the biocomposite films produced with PHBV and 10% (*w*/*w*) of functionalized arabinoxylan from the standard;
- PHBV/4 B-AX(BSG): the biocomposite films produced with PHBV and 4% (*w*/*w*) of functionalized arabinoxylan extracted from BSG.

### 2.3. Characterization of Biocomposites

#### 2.3.1. Mechanical and Morphological Properties

Neat PHBV and the different biocomposite films were prepared according to standardized dimensions specified by relevant testing standards (i.e., ASTM D882) [39]. Specimens were cut (150 mm × 4 mm × 180 μm) with a gauge length of 100 mm, and the tensile test was performed in displacement control with a crosshead speed of 10 mm/min using a Zwick/Roell Z010 tensile machine equipped with a 100 N load cell (Zwick/Roell, Ulm, Germany). The neat PHBV and biocomposite films were analyzed in triplicate.

The fracture morphology of PHBV and the biocomposite films was investigated by scanning electron microscopy (FE-SEM) using a Mira3 by Tescan (Brno, Czech Republic). PHBV and the biocomposite films were coated with gold before SEM analysis.

#### 2.3.2. Thermal Characterization of Biocomposites

The thermal behavior of PHBV and the biocomposites was investigated by differential scanning calorimetry (DSC). The specimens were analyzed using a DSC 214 Polyma (NETZSCH, Selb, Germany) in nitrogen flow. The thermal program was set for heating from −40 °C to 200 °C (10 °C/min), and for cooling from 200 °C to −40 °C (10 °C/min). PHBV and the biocomposite films were analyzed in duplicate.

The degree of crystallinity (X_c_) of the neat PHBV and biocomposite films was calculated according to Equation (1):

(1) $$X_{c}(\%)=\frac{{\Delta H}_{m}\left(\mathrm{PHBV}\right)}{{\Delta H}_{m}^{0}\left(\mathrm{PHBV}\right)}\cdot \frac{100}{w}$$

where ${\Delta H}_{m}\left(\mathrm{PHBV}\right)$ is the experimental melting’s enthalpy of the sample (J/g), ${\Delta H}_{m}^{0}\left(\mathrm{PHBV}\right)$ represents the melting’s enthalpy of 100% crystalline PHBV (146 J/g) [40,41], and w is the weight of PHBV in the formulations of biocomposites.

Furthermore, thermogravimetric (TGA) and derivative thermogravimetric (DTG) analyses were carried out on PHBV and the biocomposite films produced. Tests were performed using a Setsys Evolution Thermo Mechanical Analyzer (Setaram, Caluire-et-Cuire, France), setting the temperature range from 25 °C to 800 °C with a heating rate of 10 °C/min in nitrogen flow. PHBV and the biocomposite films were analyzed in duplicate.

## 3. Results and Discussion

### 3.1. Synthesis of Benzoate-Functionalized Arabinoxylans

Initially, a model compound of arabinoxylan-bound benzoate was prepared using standard arabinoxylan, following a previously established procedure [31,35]. This served as a baseline to better understand the behavior of functionalized arabinoxylan derivatives. Subsequently, the benzoate-modified arabinoxylan derivative was extracted from BSG using a patented fractionation protocol. The modification process involved esterifying the hydroxyl groups of arabinoxylans’ repeating units with a benzoic acid congener (Figure S1). The resulting derivative achieved an extraction efficiency of 81% and ~90% for the arabinoxylans recovered from BSG and arabinoxylan standard, respectively [31]. The extraction yield was calculated based on the residual arabinoxylans detected in the liquid fraction, as described in the Supplementary Information.

The structural modification was confirmed through ATR-FTIR spectroscopy, which revealed a reduction in the 3100–3600 cm^−1^ region, indicative of the replacement of hydroxyl groups with benzene groups from the benzoate congener. Additionally, ester bond formation was confirmed by the presence of peaks at 3070, 1720, 1260, and 710 cm^−1^, which were absent in the spectra of standard arabinoxylans (Figure S2). These results demonstrate successful functionalization.

### 3.2. Biocomposite Characterization

#### 3.2.1. Mechanical Properties and Morphology of Biocomposites

**Figure 1:**
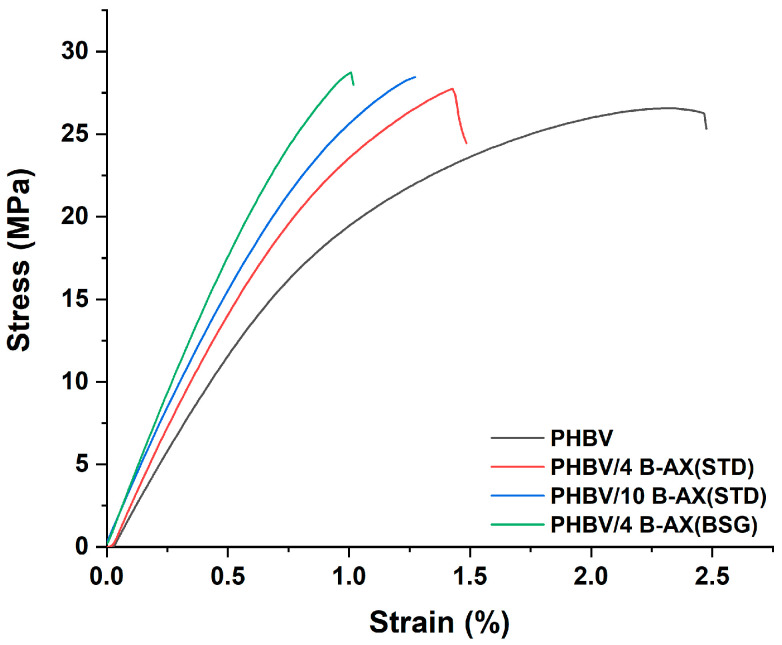
Typical stress vs. strain curves for neat PHBV and PHBV/AX composites.

The mechanical properties, including tensile strength, Young’s modulus, and elongation at break, were determined under tension for all formulations. The thickness of the biocomposite films produced was around 180 μm. The typical tensile stress–strain curves of PHBV, PHBV/4 B-AX(STD), PHBV/10 B-AX(STD), and PHBV/4 B-AX(BSG) are illustrated in Figure 1, while Table 1 provides the average values of tensile strength, Young’s modulus, and elongation at break obtained from the stress–strain curves of each formulation. Numerous studies have been carried out to evaluate the impact of using food waste-derived fillers (e.g., coffee silverskin, wheat straw fibers, oat hull fibers, olive pomace, and vine shoots) on the mechanical properties of PHBV or PHA polymer matrices [42,43,44]. The use of these fillers often resulted in reduced tensile strength and Young’s modulus. In particular, the lower value of tensile strength is usually ascribed to several factors such as poor filler dispersion and interfacial adhesion with the polymer matrix [45,46,47,48]. Strategies such as optimizing the processing parameters and modifying filler surfaces, as demonstrated in this work, can help mitigate the negative effects on tensile strength and improve the overall composite performance. The results of this work showed that the addition of 4 and 10% B-AX(STD) to the PHBV polymer matrix resulted in an increased tensile strength compared to neat PHBV (25.5 ± 1.4 MPa). In particular, the formulation with 4% B-AX(STD) showed the highest tensile strength (28.7 ± 1.5 MPa), while a plateau as the filler percentage increased up to 10% was detected (28.2 ± 0.6 MPa). It is interesting to note that the filler also led to a significant increase in Young’s modulus (~20%). The polymer film without a filler (PHBV) exhibited brittle behavior with limited plastic deformability and toughness, which were further reduced as the filler percentage increased. Considering that a 10% amount did not appear to improve the mechanical performance, it was decided to produce only a formulation with 4% by weight of arabinoxylan derived from brewers’ spent grain. In this case, the modulus compared to the analogous formulation with standard arabinoxylan increased by another 20% while maintaining the same tensile strength, suggesting that the addition of functionalized arabinoxylan enhanced both the tensile strength and Young’s modulus of the neat PHBV. The addition of a rigid filler can enhance the stiffness of a polymer [49,50] because it restricts the mobility of macromolecules. However, the filler itself cannot deform under external stress in the same manner as the polymer, as it exhibits elastic behaviors completely different from those of the surrounding polymer. Consequently, it may act as a stress concentrator [51]. The addition of the present filler to PHBV does not appear to interfere with stress transfer at the interface to an extent that would result in a reduction in tensile strength. The concentration of 4% (*w*/*w*) functionalized arabinoxylan contributes to greater improvements in the mechanical properties, even though the difference in tensile strength at 10% by weight is marginal and could be explained by the non-homogeneity of filler dispersion above certain concentrations.

**Figure 2:**
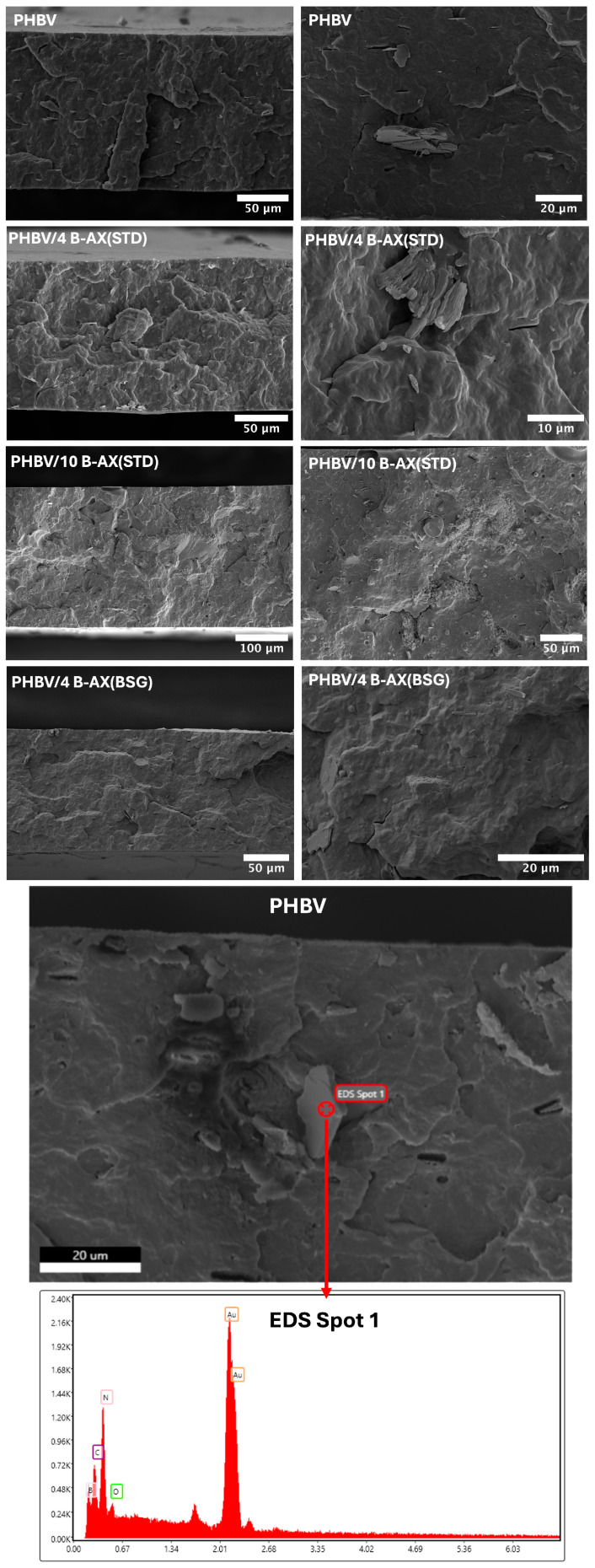
SEM micrographs detailing the fracture surface of all PHBV-based formulations and EDS analysis of neat PHBV.

The brittle behavior of the examined films is also evident from the analysis of the fracture surfaces observed at the end of the tensile tests. SEM micrographs for all formulations are included in Figure 2. The micrographs confirm the absence of macroscopic plastic deformation, showing a fracture surface that becomes more jagged following the addition of the filler, which can interfere with crack propagation. At a lower filler concentration (4%), the fracture surface indicates a homogeneous and uniform dispersion of the filler within the PHBV polymer matrix, suggesting good interfacial adhesion between the arabinoxylan particles and the PHBV polymer matrix. These features indicate a suitable compounding process and compatibility between the polymer matrix and the filler, facilitating a uniform distribution and minimizing the agglomeration of the arabinoxylan particles. In contrast, SEM analysis of composites with a higher filler amount (10%) revealed a heterogeneous surface morphology characterized by irregularities, voids, and agglomerates. The presence of heterogeneous spots on the surface fracture suggests the difficulty of achieving uniform dispersion and distribution of the filler within the PHBV polymer matrix, thereby confirming the leveling off of the tensile mechanical properties [52]. The morphology of the samples also revealed the presence of lamellar fillers, which, as shown by the EDS analysis in Figure 2, were found to be composed of nitrogen and boron. These are boron nitride fillers, which are added to the base polymer because they act as nucleating agents and facilitate its crystallization [53,54,55].

#### 3.2.2. Thermal Properties of Biocomposites

**Figure 3:**
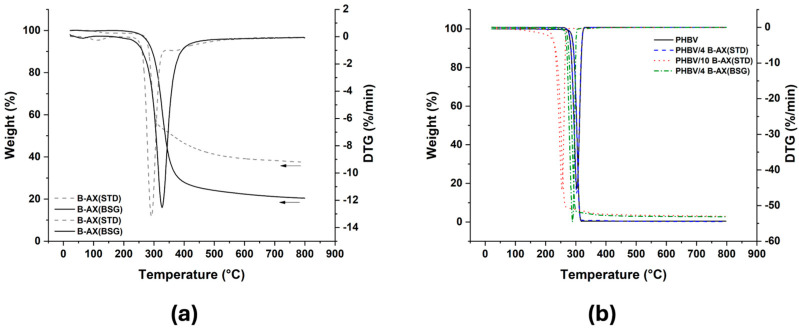
TGA curves and derivative weight against temperature (**a**) of the fillers and (**b**) of the PHBV-based composites.

TGA and DTG curves obtained under nitrogen atmosphere for PHBV, PHBV/4 B-AX(STD), PHBV/10 B-AX(STD), PHBV/4 B-AX(BSG), and neat fillers are shown in Figure 3. The temperatures of the maximum rate of decomposition (T_max_) of the biocomposites, as well as the temperatures at 5% (T_5%_) and 10% (T_10%_) weight loss, were evaluated and are summarized in Table 2. T_max_ represents the temperature at which the sample reaches the maximum decomposition rate, as indicated by the peak in the DTG curve. In the context of biocomposites, T_max_ can provide information on the temperature range at which significant degradation of the polymer matrix and reinforcement occurs. T_5%_ is particularly useful for comparing the thermal stability of different materials and assessing their sensitivity to degradation at lower temperatures. Generally, the addition of filler-derived food waste (e.g., coffee silverskin, wheat straw fibers, and vine shoots) suddenly reduces the T_max_ and T_5%_ [42,44,46]. In this case, the same value of T_max_ (303 °C) for the neat PHBV and PHBV/4 B-AX(STD) indicates that the addition of 4% B-AX(STD) does not significantly affect the maximum decomposition rate. However, the lower T_max_ value (287 °C) observed for PHBV/10 B-AX(STD) suggests that the higher concentration (10% *w*/*w*) of B-AX(STD) may influence the decomposition behavior of the biocomposite, potentially leading to a reduced maximum decomposition rate and slightly lower thermal stability compared to neat PHBV.

The T_5%_ values indicate that the addition of B-AX(STD) does not significantly affect the onset of decomposition for PHBV/4 B-AX(STD) (289 °C) compared to neat PHBV (288 °C). However, the lower T_5%_ observed for PHBV/10 B-AX(STD) (269 °C) indicates that the higher concentration (10% *w*/*w*) of B-AX(STD) may lead to an earlier initiation of thermal degradation compared to neat PHBV. It is interesting to note that the filler derived from BSG imparts to the composite a thermal stability very similar to that of the standard filler, confirming the potential of this food waste. Single-step thermal degradation was observed for both the neat PHBV and PHBV/AX composites. The thermal degradation of PHBV is attributed to a random one-step β-elimination chain scission reaction [56]. Improved thermal stability of polymers has been associated with the homogeneous dispersion of fillers at low concentrations, but the clustering of particles and the formation of voids both within and around these clusters, observed at higher concentrations by SEM, might promote the permeation and diffusion of degradation products. Jaguey-Hernández et al. (2023) provided a detailed thermogravimetric analysis of arabinoxylan films, showing distinct stages of mass loss of arabinoxylan related to water evaporation (113–174 °C) and depolymerization (215–350 °C) [27]. The lower thermal stability of biocomposites with the highest concentration of the filler (10%, *w*/*w*) might be attributed to its thermal degradation, which can release acids (benzoic acid, formic acid, acetic acid, etc.) acting as catalysts for the degradation of PHBV [57]. At higher concentrations, the degradation by-products of the filler likely accumulate and interact with the matrix, reducing its thermal stability [58,59]. Following this logic, the increased filler content in PHBV/10 B-AX(STD) and the accumulation of volatile degradation products may interfere with PHBV, leading to autocatalytic reactions that accelerate the degradation of PHBV and compromise its thermal stability.

**Figure 4:**
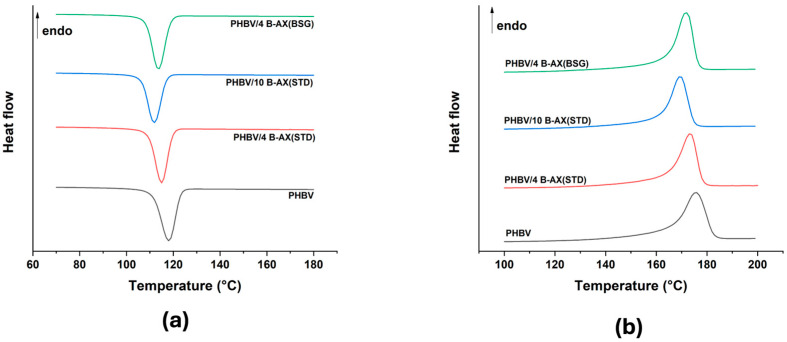
DSC thermograms of neat PHBV and PHBV-based composites during (**a**) cooling run and (**b**) second heating run.

DSC cooling and heating scans of PHBV, PHBV/4 B-AX(STD), PHBV/10 B-AX(STD), and PHBV/4 B-AX(BSG) are shown in Figure 4a and Figure 4b, respectively. This analysis was performed to evaluate the effect of functionalized arabinoxylan on the crystallization behavior of the biocomposites. The data obtained from DSC, including the melting temperature, crystallization temperature, and degree of crystallinity, are summarized in Table 3. The melting temperature (T_m_) indicates the temperature at which a crystalline polymer undergoes a phase transition from a solid to a molten state upon heating. Semicrystalline polymers, such as PHBV, typically exhibit a distinct endothermic peak corresponding to the melting process in DSC thermograms.

T_m_ provides insights into the thermal stability and processing behavior of the polymer, as well as its potential applications in processes involving melting and solidification. PHBV/4 B-AX(STD) and PHBV/10 B-AX(STD) resulted in T_m_ values of 173.4 and 169.5 °C, respectively. The reduction in T_m_ following the addition of B-AX(STD), compared to the T_m_ of neat PHBV (175.6 °C), indicates that this addition affects the crystallization behavior of neat PHBV. Lower T_m_ values indicate a reduction in the melting temperature of the crystalline regions [42,43,44] likely due to the melting of unstable imperfect crystals, and this phenomenon occurred in all formulations. The crystallization temperature (T_c_) represents the temperature at which a polymer melt undergoes a phase transition from the molten state to a crystalline solid upon cooling. Crystallization in polymers occurs through nucleation and growth of crystalline domains, leading to the formation of ordered structures. All formulations resulted in slightly lower T_c_ values compared to the neat PHBV (118.1 °C), suggesting that the addition of functionalized arabinoxylan affects the nucleation and growth of crystalline domains in PHBV. Lower T_c_ values indicate a delayed onset of crystallization [42,43,44], but a global nucleating effect on PHBV was recorded. In particular, AX, when well dispersed at concentrations below 10 wt.%, can serve as a nucleating agent, significantly enhancing nucleation. Conversely, PHBV/AX at a concentration of 10 wt.% exhibited agglomeration, which diminished the nucleating effect and reduced crystallinity compared to the formulations with a lower filler amount [42,43,44,60].

## 4. Conclusions

In this study, arabinoxylans extracted and functionalized from brewer’s spent grain was evaluated as filler for a PHBV polymer matrix at varying concentrations. The biocomposites were characterized in terms of mechanical, morphological, and thermal properties and compared to neat PHBV. The incorporation of functionalized arabinoxylans significantly enhanced the mechanical properties of the PHBV matrix, as evidenced by the increased tensile strength and Young’s modulus. These enhancements indicate that functionalized arabinoxylans effectively reinforce PHBV, improving its structural integrity and strength, which are essential for packaging applications.

SEM analysis revealed good dispersion and adhesion of the filler within the PHBV polymer matrix at lower concentrations (4% *w*/*w*), although these characteristics were less pronounced at higher filler concentrations (10% *w*/*w*). The thermal stability of the biocomposites was comparable to that of neat PHBV, with only slight variations observed in the onset temperature of thermal degradation. However, this is not true for the concentration of 10 wt.% due to the potential accumulation of large amounts of volatile degradation products, which may interfere with PHBV, leading to autocatalytic reactions that accelerate the degradation of PHBV and compromise its thermal stability.

The addition of functionalized arabinoxylans also influenced the crystallization behavior of PHBV, exhibiting a nucleating effect despite introducing crystal imperfections. This phenomenon was particularly pronounced at 10 wt.% of B-AX due to filler agglomeration, resulting in a reduced nucleating effect and lower crystallinity compared to the lower filler concentrations.

The enhanced mechanical properties, combined with the biocompatibility and sustainability of PHBV and functionalized arabinoxylans, provide the resulting biocomposites with significant promise for use in packaging applications. The improved tensile strength and stiffness offer advantages in terms of packaging durability, ensuring the protection and integrity of packaged goods during storage and transportation.

PHBV/4 B-AX(STD) and PHBV/4 B-AX(BSG) produced the best blends in terms of mechanical, morphological, and thermal properties. Notably, the brewers’ spent grain-derived filler (B-AX(BSG)) demonstrated similar advantages to the standard arabinoxylan filler (B-AX(STD)), highlighting its potential as a sustainable and effective alternative. The performance of the PHBV/4 B-AX(BSG) composite was equivalent to that of the PHBV/4 B-AX(STD) formulation, confirming that brewers, spent grain-derived arabinoxylan offers comparable reinforcement and enhances the crystallization behavior of PHBV. This finding underscores the viability of utilizing food waste-derived fillers, such as brewers’ spent grain, to achieve high-performance biocomposites while also promoting sustainability in material production.

From a sustainability perspective, there is potential for a small-scale biorefinery utilizing approximately 5000 tons of brewers’ spent grain annually—from a production of about 250,000 hL of beer per year—to produce around 4000 tons of composite formulations based on functionalized arabinoxylans.

Future work should focus on optimizing the filler concentration and processing parameters to maximize the benefits of functionalized arabinoxylans while addressing challenges related to surface heterogeneity at higher concentrations. Further studies on the long-term durability, biodegradability, and environmental impact of these biocomposites will be crucial for assessing their suitability for sustainable packaging. Additionally, exploring the potential of functionalized arabinoxylans to improve barrier, antimicrobial, and UV properties could further enhance the effectiveness of these biocomposites in food packaging applications.

## Figures and Tables

**Table 1:** Summary of tensile properties for PHBV-based composites.

Formulation	Tensile Strength (MPa)	Young’s Modulus (GPa)	Elongation at Break (%)
PHBV	25.5 ± 1.4	2.7 ± 0.1	2.3 ± 0.1
PHBV/4 B-AX(STD)	28.7 ± 1.5	3.2 ± 0.1	1.4 ± 0.1
PHBV/10 B-AX(STD)	28.2 ± 0.6	3.2 ± 0.1	1.3 ± 0.1
PHBV/4 B-AX(BSG)	28.8 ± 0.6	3.8 ± 0.1	1.1 ± 0.1

PHBV = Poly(3-Hydroxybutyrate-co-3-Hydroxyvalerate); PHBV/4 B-AX(STD) = biocomposite with 4% (*w*/*w*) addition of benzoate-functionalized arabinoxylan from standard material; PHBV/10 B-AX(STD) = biocomposite with 10% (*w*/*w*) addition of benzoate-functionalized arabinoxylan from standard material; PHBV/4 B-AX(BSG) = benzoate-functionalized arabinoxylan from brewer’s spent grains.

**Table 2:** Summary of characteristic thermal degradation temperatures of the PHBV and PHBV-based composites.

Formulation	T_max_ (°C)	T_5%_ (°C)	T_10%_ (°C)
PHBV	303	288	292
B-AX(STD)	292	277	283
B-AX(BSG)	327	278	294
PHBV/4 B-AX(STD)	303	289	293
PHBV/10 B-AX(STD)	287	269	274
PHBV/4 B-AX(BSG)	289	273	276

PHBV = Poly(3-Hydroxybutyrate-co-3-Hydroxyvalerate); PHBV/4 B-AX(STD) = biocomposite with 4% (*w*/*w*) addition of benzoate-functionalized arabinoxylan from standard material; PHBV/10 B-AX(STD) = biocomposite with 10% (*w*/*w*) addition of benzoate-functionalized arabinoxylan from standard material; PHBV/4 B-AX(BSG) = benzoate-functionalized arabinoxylan from brewer’s spent grains.

**Table 3:** The melting and crystallization temperatures (T_m_ and T_c_) and the crystallinity (X_c_) of the neat PHBV and PHBV/AX composites obtained by DSC.

Formulation	T_m_ (°C)	T_c_ (°C)	X_c_ (%)
PHBV	175.6	118.1	59.9
PHBV/4 B-AX(STD)	173.4	115.1	66.0
PHBV/10 B-AX(STD)	169.5	111.9	64.9
PHBV/4 B-AX(BSG)	171.7	113.6	67.6

PHBV = Poly(3-Hydroxybutyrate-co-3-Hydroxyvalerate); PHBV/4 B-AX(STD) = biocomposite with 4% (*w*/*w*) addition of benzoate-functionalized arabinoxylan from standard material; PHBV/10 B-AX(STD) = biocomposite with 10% (*w*/*w*) addition of benzoate-functionalized arabinoxylan from standard material; PHBV/4 B-AX(BSG) = benzoate-functionalized arabinoxylan from brewer’s spent grains.

## Data Availability

Data are contained within the article or Supplementary Materials.

## Supplementary Materials

The following supporting information can be downloaded at https://www.mdpi.com/article/10.3390/polym17010114/s1: Figure S1: Synthesis of arabinoxylan-bound benzoate; Figure S2: FTIR-ATR spectra of standard arabinoxylan and benzoate-arabinoxylans. References [31,33,35] are cited in the Supplementary Materials.
