# Supplementary material for: Brewers’ Spent Grain-Derived Arabinoxylan as a Sustainable Filler for Enhanced PHBV Biocomposites
Source: Polymers (Basel). 2025 Jan 5;17(1):114. doi: 10.3390/polym17010114 (PMC11723018; doi:10.3390/polym17010114)
Supplement: Supplementary file 1 [file polymers-17-00114-s001.zip › polymers-3407654-supplementary.pdf]

Supplementary Material

Brewers' spent grain-derived arabinoxylan as a sustainable filler for enhanced PHBV biocomposites

Ilary Belardi [1], Fabrizio Sarasini [2,*], Jacopo Tirillò [2], Pietro Russo [3], Giovanni De Francesco [1], Ombretta Marconi [1,4] and Assunta Marrocchi [5,*]

[1] Department of Agricultural, Food and Environmental Sciences, University of Perugia, 06121 Perugia, Italy; ilary.belardi@dottorandi.unipg.it (I.B.); giovanni.defrancesco@unipg.it (G.D.F.); ombretta.marconi@unipg.it (O.M.)
[2] Department of Chemical Engineering Materials Environment and UdR INSTM, University of Rome La Sapienza, 00184 Rome, Italy; jacopo.tirillo@uniroma1.it
[3] Institute of Polymers, Composites and Biomaterials, National Research Council, 80078 Pozzuoli, Italy; pietro.russo@ipcb.cnr.it
[4] Italian Brewing Research Centre (CERB), University of Perugia, 06126 Perugia, Italy
[5] Department of Chemistry, Biology and Biotechnology, University of Perugia, 06123 Perugia, Italy
* Correspondence: fabrizio.sarasini@uniroma1.it (F.S.); assunta.marrocchi@unipg.it (A.M.)

Arabinoxylan-bound benzoate from standard arabinoxylan

Standard arabinoxylans (0.25 g) were introduced into a two-necked round-bottom flask. Subsequently, 25 mL of NaOH (5% w/w) was added, and the system was maintained under magnetic stirring at a temperature of 30°C for 30 minutes. Benzoyl chloride (1.1 mL) was added dropwise to the resulting solution, maintaining the temperature at 30°C. The reaction mixture was stirred magnetically for an additional hour. After the reaction time had completed, the resulting white solid was filtered under vacuum using a sintered glass funnel, washed with ethanol, and dried. The product (Figure S1) was obtained with a yield of 81%.

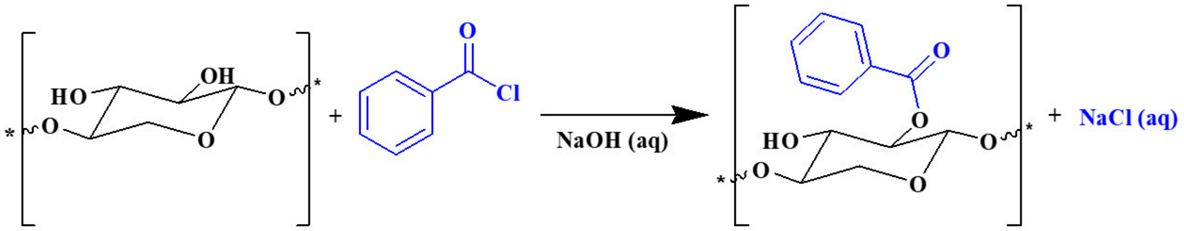

Figure S1. Synthesis of arabinoxylan-bound benzoate.

Arabinoxylan-bound benzoate from Brewers' spent grain

Wet BSGs were fractionated to obtain arabinoxylan-bound benzoate according to a patented protocol [1]. The process involves sequential extraction steps under mild alkaline conditions. First, the solid fiber fraction was isolated from the original matrix by aqueous NaOH treatment at a temperature between 50 and 70 °C. Next, the fiber fraction was treated with an aqueous alkali for at least 6 h at a temperature in the range of 30-50 °C (fiber-to-liquid ratio = 1:25–1:150 w/v; fiber-to-alkali ratio = 1:3–1:5 w/w). The obtained slurry was cooled and centrifuged to recover a solid cellulose-rich fraction, and a liquid fraction containing arabinoxylans and lignin. The addition of benzoyl chloride to the liquid fraction allowed the selective separation of solid arabinoxylan-bound benzoate (5–6%, mol/w_{AX}) (Figure S1) from lignin (T = 30–50 °C; t = 30–60 min). Yield = 89%.

The yield of arabinoxylan-bound benzoate was calculated following Eq. S1:

$$\text{Yield}(\%) = \frac{AX_i - AX_{liquid}}{AX_i} \cdot 100 \qquad \text{(Eq. S1)}$$

where AX_{liquid} represents the content of residual arabinoxylan in the liquid phase, and AX_i is the initial amount of arabinoxylans. The arabinoxylan content was determined according to the method described by Marconi et al. (2020) [2].

FTIR-ATR measurements

Fourier-transform infrared spectroscopy (FTIR) in attenuated total reflection (ATR) was used to evaluate the change in the arabinoxylan structure during functionalization (Figure S2) [3]. FTIR-ATR spectroscopy was performed using a compact FTIR spectrometer (mod. Alpha, Bruker Optics, Ettlingen, Germany) equipped with an ATR module (mod. Platinum, Bruker Optics, Germany) with a single-reflection diamond crystal. OPUS 7.5 Bruker Optics software was employed for the acquisition and analysis of the IR spectra. These were recorded in the 400–5000 cm^{-1} range averaging over 30 scans, with a resolution of 2 cm^{-1}. The spectra were corrected using the "atmospheric compensation" and baseline routines implemented in the OPUS 7.5 program.

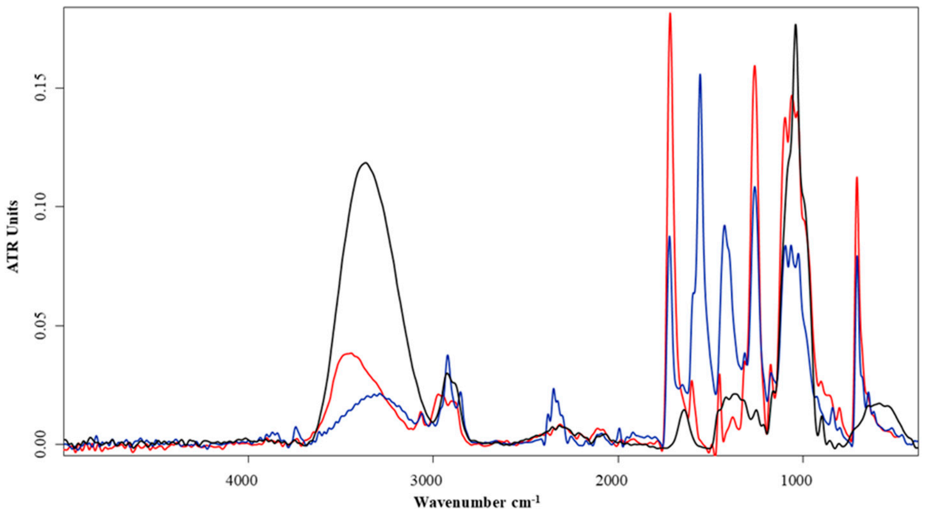

Figure S2. FTIR-ATR spectra of standard arabinoxylan (black line), benzoate-arabinoxylan from standard (red line), and BSG (blue line).

References

1. Marconi O., Marrocchi A. "Process for treating of brewing industry by-products" PCT Int. Appl. (2023), WO2023012841 A1 09-02-2023

2. Marconi, O.; Tomasi, I.; Sileoni, V.; Bonciarelli, U.; Guiducci, M.; Maranghi, S.; Perretti, G. Effects of Growth Conditions and Cultivar on the Content and Physiochemical Properties of Arabinoxylan in Barley. J Agric Food Chem 2020, 68, 1064–1070.

3. Belardi, I.; Marrocchi, A.; Alfeo, V.; Sileoni, V.; De Francesco, G.; Paolantoni, M.; Marconi, O. Sequential Extraction and Attenuated Total Reflection–Fourier Transform Infrared Spectroscopy Monitoring in the Biorefining of Brewer's Spent Grain. Molecules 2023, 28(24), 7992.
